
JOURNAL INFORMATION
==============================
NLM Title Abbreviation: Wirtschaftsdienst
Journal ID: Wirtschaftsdienst
NLM Journal ID: 101086612

Wirtschaftsdienst (Hamburg, Germany : 1949)

ISSN: 0043-6275

ARTICLE INFORMATION
==============================
PMCID: PMC7813608
PMID: 33487763
DOI: 10.1007/s10273-021-2809-5
Article ID: 2809
Article version: 1
Subjects: Leitartikel

Staatsverschuldung nach Corona: Rückkehr zur Goldenen Regel

Public debt after Corona: Return to the Golden Rule

Breuer Christian c.breuer@zbw.eu

Redaktion Wirtschaftsdienst, ZBW - Leibniz-Informationszentrum Wirtschaft, Neuer Jungfernstieg 21, 20354 Hamburg, Deutschland
Electronic publication date: 2021 Jan 19
Print publication date: 2021
Volume: 101
Issue: 1
First page: 2
Last page: 3
Copyright: © Der/die Autor(en) 2021
License: Open Access: Dieser Artikel wird unter der Creative Commons Namensnennung 4.0 International Lizenz (https://creativecommons.org/licenses/by/4.0/deed.de) veröffentlicht.
Open Access wird durch die ZBW — Leibniz-Informationszentrum Wirtschaft gefördert.
License URL: https://creativecommons.org/licenses/by/4.0/

issue-copyright-statement: © The Author(s) 2021

==============================

Die Corona-Krise hat die öffentliche Neuverschuldung explodieren lassen, weshalb die deutsche Schuldenbremse und die europäischen Fiskalregeln aktuell ausgesetzt sind. Ihre absehbare Wiedereinführung wird im Lichte der lang anhaltenden Niedrigzinsphase zunehmend kritisch diskutiert. Das unabhängige European Fiscal Board (2020) bei der Europäischen Kommission fordert eine Vereinfachung der Fiskalregeln, wobei man sich an einem Schuldenziel orientieren und Investitionen schützen sollte. Blanchard et al. (2020) plädieren für die Abschaffung der Regeln zugunsten von „fiskalischen Standards", um der Komplexität der Diskussion um Schuldentragfähigkeit gerecht zu werden. Mit ähnlicher Stoßrichtung fordern Hüther und Südekum (2020) in Deutschland eine „flexible" Schuldenbremse sowie einen staatlichen „Vermögenshaushalt".

Die Diskussion wird vor dem Hintergrund der andauernden Niedrigzinsphase geführt, die auch die Bewertung von Staatsverschuldung beeinflusst (Krämer und von Weizsäcker, 2020). Da das Verhältnis von Zinssatz r und Wachstumsrate g kleiner als 1 ist, bringt Staatsverschuldung keine fiskalischen Kosten mit sich (Blanchard, 2019). Wäre der Zins größer als die Wachstumsrate ($r>g$), würde der Zähler der Staatsschuldenquote (wegen Zins und Zinseszins) schneller steigen als der Nenner (durch Wachstum und Inflation), wenn dieser Anstieg nicht durch Primärüberschüsse kompensiert würde. Bei niedrigen Zinsen bzw. $r<g$ bildet sich hingegen die Quote aus Staatsverschuldung und Bruttoinlandsprodukt (BIP) von selbst zurück. Sowohl in den USA als auch in Deutschland, Frankreich und Italien ist $r<g$ bei Staatsanleihen die historische Norm (Blanchard, 2019 und Barro, 2020).

Dieser Zusammenhang wurde bei der Einführung der Schuldenbremse vor zehn Jahren anders gesehen. Nach den inflations- und zinstreibenden Ereignissen der 70er, 80er (Ölpreiskrisen) und 90er Jahre (Wiedervereinigung), trugen in der Finanzkrise steigende Risikoaufschläge auf Staatsanleihen zu der Sorge bei, dass Staatsverschuldung bei hohen Zinsen ($r>g$) fiskalische Lasten mit sich bringen würde. Dies erklärt den damals vorherrschenden Ansatz, Fiskalregeln eng zu gestalten, Defizit- und damit Schuldenquoten auf niedrigem Niveau zu begrenzen sowie Ausnahmen für Investitionen oder konjunkturelle Impulse nicht zuzulassen.

Viele aktuelle Reformvorschläge teilen die Sicht, dass Investitionen bevorzugt zu behandeln sind. Insofern bietet sich die Rückkehr zur Goldenen Regel an, wie sie bis 2010 in Artikel 115 des Grundgesetzes verankert war. Diese erlaubte die Nettokreditaufnahme in Höhe der staatlichen Bruttoinvestitionen und knüpfte insofern systematisch an das staatliche Sparen an. Für die Goldene Regel sprechen drei zentrale Argumente:

*Erstens*, das Defizit als Zielgröße der Fiskalregeln reduziert Anreize, in Zukunftsprojekte zu investieren. Gerade weil die Fiskalregeln im Interesse der kommenden Generationen begründet werden, spricht wenig dafür, Ausgaben für Zukunftsinvestitionen zu begrenzen. Tatsächlich sind die Nettoinvestitionen in den Peripherieländern Europas seit langem negativ, sodass die öffentliche Infrastruktur verschleißt, was auch über längere Zeit in Deutschland zu beobachten war. Kritik der klassischen Goldenen Regel verweist darauf, dass neben den Bau- und Anlageinvestitionen auch andere investive Ausgaben sowie entsprechende Abschreibungen berücksichtigt werden sollten. Da es aber bisher an einem konsensfähigen und praktikablen alternativen Investitionsbegriff mangelt, empfiehlt sich aus rein pragmatischen Gründen die klassische Goldene Regel unter Verwendung der staatlichen Bruttoinvestitionen (oder einer Kombination aus Defizitziel plus Nettoinvestitionen), was zunächst Sachinvestitionen begünstigen und Erhöhungen heute relevanter Zukunftsinvestitionen, z. B. in die Digitalisierung sowie die Energie- und Verkehrswende, erlauben würde. Dabei wird der zusätzliche Investitionsbedarf in Deutschland auf jährlich rund 45 Mrd. Euro (1,3 % des BIP) über die kommenden zehn Jahre geschätzt.

*Zweitens*, der Zielwert der klassischen Goldenen Regel für die Begrenzung der Schuldenquote ist pragmatisch. Die neuen Defizitbeschränkungen der Schuldenbremse seit 2010 (0,35 % des BIP) und des Fiskalpakts (0,5 bzw. 1 % des BIP) sind erheblich enger als die Maastricht-Obergrenze von 3 %. Sie ließen die Schuldenquote beim Verhältnis aus Defizitquote und Wachstumsrate konvergieren, was bei einem Nominalwachstum von ca. 3½ % eine Schuldenquote von 10 % bis 15 bzw. 30 % ergibt. Für höhere Zielwerte spricht neben dem neu zu bewertenden Zinslastargument bei $r<g$, dass durch Staatsanleihen auch sichere Vermögenswerte angeboten werden, an denen es mangelt (Caballero und Farhi, 2017). Daher ist fraglich, ob eine schnelle und schmerzhafte Rückführung der Schuldenquoten in Westeuropa von deutlich höheren Werten (ca. 100 bis 140 %) sinnvoll, angemessen und vermittelbar ist. Die klassische Goldene Regel würde hingegen Defizite in Höhe der staatlichen Bruttoinvestitionen erlauben, was heute in Deutschland den geschätzten Abschreibungen (ca. 2,1 % des BIP) entspricht. Dies würde bei einem Nominalwachstum von 3½ % die Bruttoverschuldung bei genau 60 % des BIP stabilisieren – dem Maastricht-Kriterium. Auch eine Erhöhung der Nettoinvestitionen würde nur den Zielwert der Bruttoschuldenquote verschieben, die relevante Nettoverschuldung aber unberührt lassen.

*Drittens*, die Fiskalregeln sollten ein gesundes Maß an Stabilisierungspolitik ermöglichen. Da Geldpolitik an der Nullzinsgrenze wenig wirkungsvoll ist, wird der Fiskalpolitik in der Liquiditätsfalle eine gestaltende Rolle zugeschrieben (Truger, 2020). Die Rückkehr zur Goldenen Regel würde antizyklische Maßnahmen in Form von Investitionsprogrammen erlauben. Dies sollte wie bisher um eine Konjunkturkomponente ergänzt werden, auch wenn die Methodik der Berechnung der konjunkturellen Defizite verbesserungsbedürftig zu sein scheint (Brooks und Fortun, 2020 und Heimberger, 2020).

Die Diskussion um die Wiedereinführung der Fiskalregeln in Deutschland und Europa sollte neben der heute veränderten Bewertung der Zinsbelastung bei $r<g$ auch die Funktion der Staatsanleihen als sichere Anlageform, zur Bekämpfung der Liquiditätsfalle sowie die Nettorendite produktiver Investitionen berücksichtigen. Aus pragmatischen Gründen bietet sich die klassische Goldene Regel an, die auf die Begrenzung der Nettoverschuldung abzielt und Investitionen im Interesse der kommenden Generationen fördert.

Christian Breuer
ZBW – Leibniz-Informationszentrum Wirtschaft
c.breuer@zbw.eu

Christian Breuer ist Chefredakteur von Wirtschaftsdienst und Intereconomics in der ZBW — Leibnizformationszentrum Wirtschaft in Hamburg.


Literatur

Barro R J NBER Working Paper 2020
Blanchard O Public Debt and Low Interest Rates American Economic Review 2019 109 4 1197 1229 10.1257/aer.109.4.1197
Blanchard O Leandro und J.^Zettelmeyer A Economic Policy 2020
Brooks R Fortun J Eurozone Output Gaps and the COVID-19 Shock Intereconomics 2020 55 5 291 296 10.1007/s10272-020-0918-9
Caballero R Farhi E The Safety Trap The Review of Economic Studies 2017 85 1 223 274
European F B Annual Report 2020 2020
Heimberger P Potential Output, EU Fiscal Surveillance and the COVID-19 Shock Intereconomics 2020 55 3 167 174 10.1007/s10272-020-0895-z 32536715 PMC7276112
Hüther M Südekum J Die Schuldenbremse nach der Corona-Krise Wirtschaftsdienst 2020 100 10 746 752 10.1007/s10273-020-2757-5
Krämer H von Weizsäcker C C Sparen und Investieren im 21. Jahrhundert: Das Ende der Kapitalknappheit Wirtschaftsdienst 2020 100 8 569 572 10.1007/s10273-020-2711-6
Truger A Reforming EU Fiscal Rules: More Leeway, Investment Orientation and Democratic Coordination Intereconomics 2020 55 5 277 281 10.1007/s10272-020-0915-z
